# State lines and species divides: Inconsistencies and opportunities in invasive species policies across the eastern United States

**DOI:** 10.1002/eap.70287

**Published:** 2026-08-02

**Authors:** Joseph Drake, E. M. X. Reed, David Haak, Bryan Brown, Meryl C. Mims, Michael G. Sorice, Haldre Rogers, Scott Salom, Todd Schenk, Jacob N. Barney

**Affiliations:** ^1^ Invasive Species Collaborative, Virginia Tech Blacksburg Virginia USA; ^2^ Southeast Alaska Watershed Coalition Juneau Alaska USA; ^3^ School of Plant and Environmental Sciences, Virginia Tech Blacksburg Virginia USA; ^4^ Department of Biological Sciences Virginia Tech Blacksburg Virginia USA; ^5^ Department of Forest Resources and Environmental Conservation Virginia Tech Blacksburg Virginia USA; ^6^ Department of Fish and Wildlife Conservation Virginia Tech Blacksburg Virginia USA; ^7^ Department of Entomology Virginia Tech Blacksburg Virginia USA; ^8^ School of Public and International Affairs, Virginia Tech Blacksburg Virginia USA

**Keywords:** biological invasions, invasive species, legislation, management, public policy, regulation, transdisciplinary research

## Abstract

Invasive species are a threat to ecological and anthropogenic systems. In the United States, policies to coordinate funding and precipitate management action have been slow to emerge at the federal level, and there is a patchwork of regulation and legislation at the state level. This means that managers and policymakers, already facing limited budgets and evolving goals for action on invasive species, also face detrimental policy inconsistencies across states. Although previous research has explored state‐level invasive plant policy, policy relating to invasive invertebrate and vertebrate taxa (and across all three) is understudied. We expand upon previous taxa‐limited examinations of public policy related to invasives, looking across all taxonomic groups, including plants, to explore coherency of state regulations. We expanded the taxonomicscope of a database of policies in 21 contiguous eastern US states and used it to examine (in)consistencies in spatial trends for invasive species listed in policies across and within taxonomic groups. We examined the coherency of neighboring states and regional overlap of named species. We also analyzed correlations between distances among states and the species listed in the policy to examine regional trends. We found 1117 policy segments relevant to invasive species with 448 naming at least one taxon at the genus or species level. Of these, 35.3% were plants, 19.9% were invertebrates, and 44.8% were vertebrates. The distribution of taxa contained within policies varied across states, underscoring high variability in the proportion of taxa listed in the policies of neighboring states. Even lower policy coherency existed at the regional scale, particularly for invertebrate and vertebrate taxa. Our results indicate that policy inconsistency exists across all taxonomic groups, and the lack of attention to invasive invertebrates and vertebrates across state policies is particularly concerning. Policy inconsistency means that proactive states are susceptible to invasion from neighboring states where invasives are not similarly regulated. There is an opportunity to improve coordination between states to reduce vulnerability to invasives due to policy inconsistency.

## INTRODUCTION

Invasive species have a wide variety of impacts, which include decreasing native species abundance and richness (Vilà et al., 2011); impairing ecosystem services (Charles & Dukes, 2008; Gallardo et al., 2024; Walsh et al., 2016); and negatively affecting human health, human wellbeing, agriculture, infrastructure, national security, tourism, and other areas of import (Roy et al., 2023). The economic costs of biological invasions to the global economy were estimated to be at least US$1.738 trillion from 1970 to 2019 (Bacher et al., 2023; Diagne et al., 2021). The cost of inaction is calculated to be much higher than the cost of potential management interventions (Branco et al., 2021; Cuthbert et al., 2022), yet proactive policies for management are often slowed, made impotent, or underfunded (Funk et al., 2014). Such delays are particularly concerning for invasive species management, as the most cost‐effective and successful strategies are those taken before species are introduced or immediately after detection. Furthermore, managers and policymakers are challenged with limited budgets and an evolving set of goals and needs to address the complex issue of invasion across local, national, and global scales (Pyšek et al., 2020).

In the United States, there is no federal legislation mandating a central authority to direct and implement invasive species detection and response (Burgos‐Rodríguez & Burgiel, 2020). As a result, a patchwork of agencies and interinstitutional entities exists to address various aspects of invasive species management. For instance, the National Invasive Species Council (NISC) is an institution in the executive branch (US) that provides high‐level leadership to help overcome barriers to communication, collaboration, and data sharing among diverse stakeholders (Barney et al., 2019; Beaury et al., 2020; Reaser, Burgiel, et al., 2020; Reed, Cathey, et al., 2023). Yet the effectiveness of entities like NISC to increase capacity for cohesive invasive species policy is undermined by barriers such as inconsistent funding and vacillating political support (Reaser, Simpson, et al., 2020; Simberloff et al., 2020).

In part, because there is no overarching federal policy that coordinates management in the US (Klizentyte et al., 2021), state‐level policies are particularly relevant to invasive species management (Reed, Cathey, et al., 2023). As a result, many policies lack a unified approach, are unenforceable, and are not reliably applied across states, which impedes efforts to coordinate prevention efforts and management responses (Beaury et al., 2021; Klizentyte et al., 2021; Lakoba et al., 2020; Reed, Cathey, et al., 2023). For example, Reed, Cathey, et al. (2023) found tremendous variation in the number of policies on invasive species among 21 eastern states, suggesting little to no coordination among neighboring states. State government funding is also inconsistent and uncoordinated. Total reported expenditures on state invasive species management efforts in the US ranged from US$28,370 in Connecticut to US$118,695,389 in Washington (Foster et al., 2024). Across states, inconsistencies in regulated taxa, regulatory authorities, and funding can act as barriers to successful prevention, control, and management as invasive species move across political boundaries (Beaury et al., 2021; Hulme, 2021a; McCubbins et al., 2013).

Recent studies suggest sizable variation in both the amount of regulation and how much is spent on the management of invasive species across US states, yet the degree to which policies vary among taxa remains largely unknown. Examinations of regional and national invasive species policies have largely focused on invasive plants (e.g., Beaury et al., 2021; Fox & Gordon, 2017; Lakoba et al., 2020; McCubbins et al., 2013; but see Doelle, 2003; Klizentyte et al., 2021). However, invasive mammals and insects are also among the costliest taxonomic groups, incurring costs over US$234 billion and US$126 billion respectively, compared to US$190 billion for plants (Fantle‐Lepczyk et al., 2022).

Here, we expand on previous work to explore the taxonomic (in)consistencies of regulatory and statutory law related to invasive species across 21 US states, which has been identified as a major need (Beaury et al., 2021; Doelle, 2003; Klizentyte et al., 2021; Lakoba et al., 2020; Reed, Cathey, et al., 2023). Addressing this need can identify incongruities in policy that result in reduced efficacy of responses and mitigation efforts (e.g., Barney et al., 2019; Beaury et al., 2021; McCubbins et al., 2013; Roy et al., 2023). We described regional patterns of codified policies (statutes and regulations) from a database of 21 contiguous eastern US states developed by Reed, Cathey, et al. (2023) and updated by our team to cover policies through 2023 (Haak & Drake, 2026). We then examined the (in)consistencies in spatial trends for species listed in policies across and within taxonomic groups. We also analyzed the degree to which species lists align among states to explore regional trends. Finally, we chose several species as “case studies” to illustrate the implications of policy inconsistencies, potential successes, and opportunities for coordinated policy efforts to manage invasive species. Individual case studies give context to the trends and gaps we identify and highlight how we can further improve coordination among partners and stakeholders in communication, data sharing, and actions.

## METHODS

### Data

We derived our data from a living database of state policies related to invasive species first published in Reed, Cathey, et al. (2023). This database contains codified policies, that is, *statutes* passed by state legislative bodies and *regulations* adopted by administrative agencies, from 21 eastern US states (Table 1; Haak & Drake, 2026). This database does not include uncodified policies, such as some noxious weed lists and agency planning documents (Beaury et al., 2021; Klizentyte et al., 2021; Lakoba et al., 2020) or those listed only on government websites (Reed, Cathey, et al., 2023). It comprehensively includes all enforceable mandates (Coupette et al., 2021) found in legal databases (e.g., Thomson Reuters Westlaw). Statutory and regulatory codes are organized hierarchically and are generally made up of titles, which are divided into chapters containing sections (the smallest policy code unit). However, states organize their codes differently, making it difficult to directly compare counts of policy sections across states (Reed, Cathey, et al., 2023). Therefore, the database uses the term *chapter* to refer to a common unit or level of policy among states (a glossary of terms can be found in Appendix S1). This approach follows the methodology of the *State RegData* data set version 4.0 (McLaughlin & Nelson, 2021). It also helps reduce variability caused by administrative and legislative differences among states and agencies within states while retaining similar levels of topic specificity.

**TABLE 1:** The total and unique (without counting the same species multiple times in separate policy chapters) records of listed species in invasive species related policy in 21 eastern US states.

State	Total species listed in chapters	Unique species listed in chapters	No. neighbors	No. unique species in neighborhood	No. in common	Among‐neighbor overlap (%)	Regional overlap (%)
Alabama	170	156	4	669	94	60.3	14.1
Connecticut	163	146	3	497	93	63.7	18.7
Delaware	157	147	3	385	75	51.0	19.5
Florida	472	345	2	233	71	20.6	30.5
Georgia	126	119	5	646	70	58.8	10.8
Kentucky	108	101	3	364	45	44.6	12.4
Massachusetts	164	128	5	650	84	65.6	12.9
Maryland	202	171	4	461	92	53.8	20.0
Maine	134	111	1	250	78	70.3	31.2
Mississippi	343	297	4	213	77	25.9	36.2
North Carolina	207	165	4	484	89	53.9	18.4
New Hampshire	510	250	3	227	115	46	50.7
New Jersey	215	182	3	447	71	39.0	15.9
New York	335	288	6	589	125	43.4	21.2
Pennsylvania	150	109	5	603	78	71.6	12.9
Rhode Island	184	179	3	416	45	25.1	10.8
South Carolina	195	151	2	249	68	45.0	27.3
Tennessee	102	92	6	741	65	70.7	8.7
Virginia	340	270	5	448	114	42.2	25.5
Vermont	81	67	3	517	54	80.6	10.5
West Virginia	92	78	4	502	50	64.1	10.0

*Note*: Among‐neighbor overlap shows the percent of invasive species in states that occur in at least one neighboring state. Regional overlap represents the percent of species that the focal state has in common with the entire neighborhood of adjacent states. These metrics show different facets of policy consistency among states.

The database has been updated with policies through 2023 (Haak & Drake, 2026). Following Reed, Cathey, et al. (2023), we used the Thomson Reuters Westlaw Database to identify policies using keyword searches related to invasive species policy and management in state statute and regulatory policy. The original dataset included 706 policy chapters extracted between May and August 2021 (Reed, Cathey, et al., 2023). We identified 411 new policy chapters in our searches between September 2021 and December 2023. These new chapters were screened to ensure keywords were present in the text and in contexts relevant to invasive species. Policy and biological data were then extracted and screened using the same workflow as described in Reed, Cathey, et al. (2023). We also extracted information pertinent to the content of the policy, including whether the policy represented a statute or regulation, which regulatory agency type was responsible for the policy implementation (if the chapter was non‐statute), the general taxa the policy referenced, and whether the taxa represented an aquatic or terrestrial pathway of invasion (Haak & Drake, 2026).

### Analysis of policy consistency across and within taxa and states

To explore the consistency of invasive species listed in policies across all taxa, we first analyzed named species within policy chapters. For each state, we compiled a list of all species named in at least one policy chapter. When a policy chapter identified a taxon at the family or genus level, we considered it to be a single entry (i.e., as a “species”) to avoid inflating the differences among states (Beaury et al., 2021; Lakoba et al., 2020). For example, Florida's regulation regarding the invasive lionfish was at the genus level (*Pterois* spp.). There are 12 recognized *Pterois* species (FACAR, 2018), but we only included the genus in the list of species named in Florida whereas both *P. miles* and *P. volitans* were specifically named in New York. If a species occurred in more than one policy chapter, we retained the earliest listing in state policies for each species or taxonomic entry. After we compiled species within policies, we grouped each entry into three high‐level taxonomic categories (plants, vertebrates, and invertebrates) to ensure there would be enough entries in each category for analysis. We note the exclusion of fungi from our analysis due to the paucity of policy chapters identified containing references to fungi. We were then able to quantify trends across all taxa and within taxonomic categories.

Species considered in this analysis largely came from the state‐level policies themselves. However, we also incorporated species defined as introduced or invasive by the US Register of Invasive and Introduced Species (US‐RIIS) version 2.0 for the contiguous United States (Simpson et al., 2022; Simpson & Eyler, 2018) and the USGS Nonindigenous Aquatic Species database (NAS; U.S. Geological Survey, 2023) into our dataset. This allowed us to examine whether discontinuities exist between species designated invasive by national registries and the total set of species that were included in state‐level policies (Reed, Cathey, et al., 2023).

We assessed the consistency of species lists across states and their adjacent neighbors using two metrics: among‐neighbor overlap and regional overlap (Beaury et al., 2021). Among‐neighbor overlap was calculated by finding the percent of a listed taxon in the focal state's policies that also occurred in at least one neighboring state's policies. Low among‐neighbor overlap suggests that the policies of the focal state and its neighbors are not coordinated. Regional overlap was calculated by dividing the species that the focal state has in common with neighboring states' policies by the total number of species identified in policies in all adjacent states. States with relatively high regional overlap values are listing proportionally more species in common with adjacent states than states with low values of regional overlap. Beaury et al. (2021) suggests that states with high among‐neighbor or high regional overlap have “good neighbors,” meaning their priorities appear to be aligned according to the overlap of invasive species within policy chapters. High levels for both among‐neighbor and regional values represent a best‐case scenario and indicate realized regional attention among neighboring states for invasive species. We calculated metrics for all taxa combined as well as for plant, invertebrate, and vertebrate taxa subsets to explore taxon‐related disparities among policy consistency.

To better understand trends beyond adjacent states, we examined the number of shared species between all possible pairwise combinations for the 21 states using distance among those states' geographic centroids. These centroids were calculated using the package *terra*, forcing geographic centroids to fall within polygon boundaries (Hijmans, 2023). We used pairwise distances for all state centroids in combination with either the number of all taxa or the subset of plants, vertebrates, and invertebrates to test for significant correlations among pairwise distances and shared species between state pairs. We tested correlation using Kendall's τ to account for the lack of independence among data points (Kendall, 1938), as it is robust to deviations of assumptions of normality, and we conducted permutation tests (*n* = 9999) to derive a probability of deviation of observed correlations relative to correlations expected by chance.

### Case studies

We explored two sets of case studies (three species each) to illustrate limitations and examples of coordinated policy adoption. The first three species are among the four that occurred in policies across all 21 states in the database. The second set of three species was selected to be emblematic of plant, invertebrate, and vertebrate taxonomic groups that we evaluated in the analyses above. Each case study illustrates various facets of invasive species policy and identifies areas where policy could be coordinated to improve regional coherency and neighborhood overlap to promote resilient invasive management strategies. To show whether a state was proactive or reactive to each case study species, we used the Global Biodiversity Information Facility (gbif.org) to pull all available occurrences of species listed in our invasive species related policy chapters (GBIF, 2024) and compared these to the earliest policy listed in each state for case study species. If a state enacted a policy chapter naming a species/taxa before or in the same year that a species was first recorded being present in the state, then that state was considered to be proactive. Otherwise, the state was considered to be reactive or may not have a policy related to that invasive species at all.

## RESULTS

### Policy overview

**FIGURE 1:**
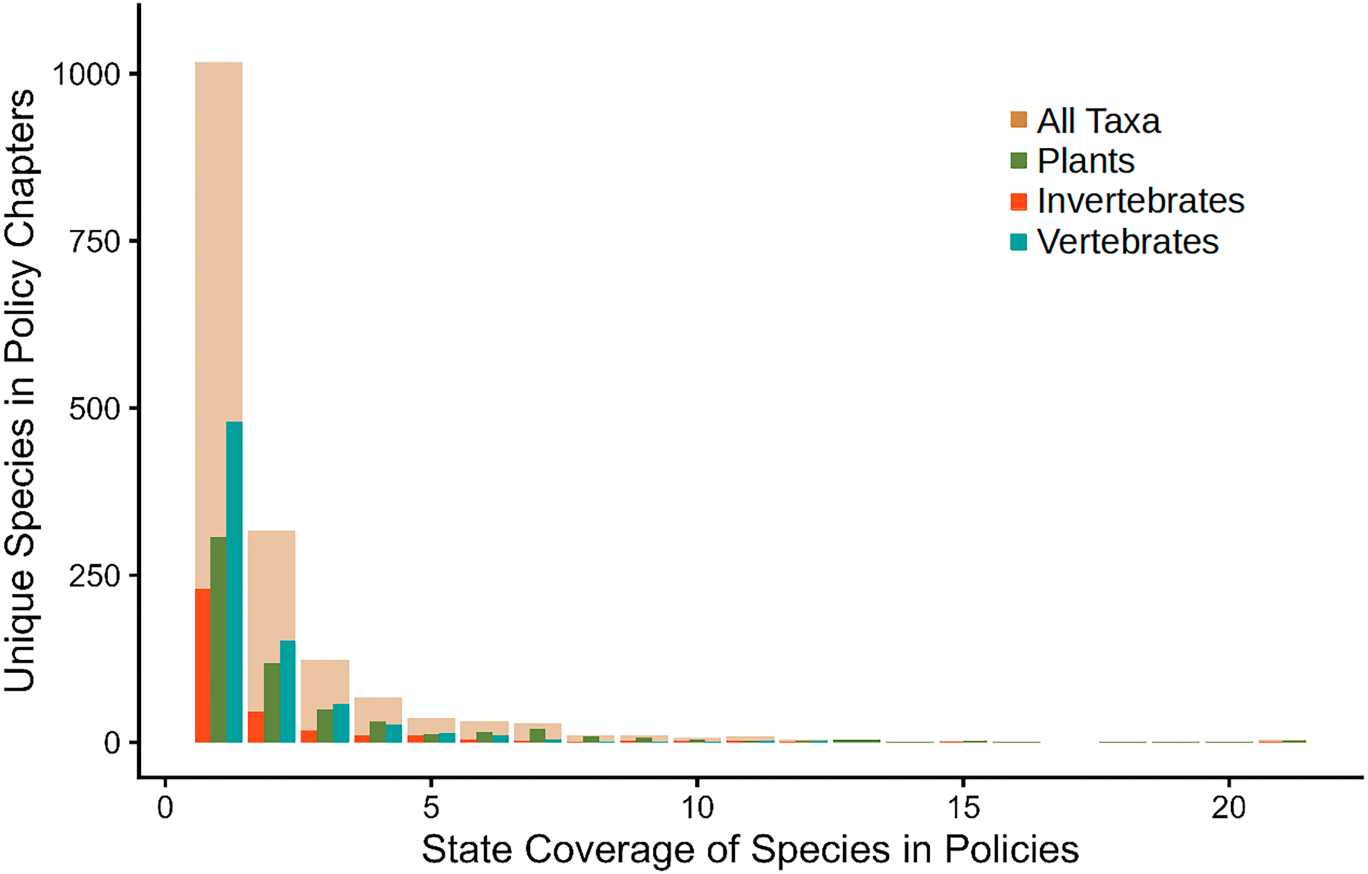
The distribution of species mentioned in policy chapters across states in the database. Most species occur in policy chapters in fewer than five states. Only four species (one listed at genus level) occur in all states within the database. Plants, generally, are listed more frequently in more states, but the total numbers are still low. Little consistency exists among the entire database.

There were 1117 policy chapters relevant to invasive species in the 21 eastern US states in our database (Haak & Drake, 2026); 411 new policies were added to the original 706 presented by Reed, Cathey, et al. (2023). Of the full dataset, 448 policy chapters named at least one taxa at the genus or species level (hereafter referred to as species). The total number of unique times species named in policy chapters within each state ranges from 81 (Vermont) to 510 (Florida). There were 1682 unique species named across all states (Table 1), ranging from 67 (Vermont) to 345 (Florida). The majority of species (1017; 60.5%) were only recorded in a single state, while 7% of listed species (119) were recorded in more than five states (Figure 1). Only four species were recorded in policy chapters across all 21 states in the database: western honeybee (*Apis mellifera*), creeping thistle (*Cirsium arvense*), quackgrass (*Elymus repens*), and dodder (*Cuscuta* spp).

When broken down by taxonomic group, 35.3% (594) of all listed species were plants, 19.9% (335) were invertebrates, and 44.8% (753) were vertebrates. The number of plants named in at least one policy within a state ranged from 20 (Rhode Island) to 161 (New York); invertebrates ranged from 2 (Kentucky) to 105 (Mississippi); and vertebrates ranged from 3 (Massachusetts) to 189 (Virginia; Appendix S1: Table S1). The number of species listed in only one policy chapter across all states was 241 (40.6%) plants, 203 (60.6%) invertebrates, and 425 (56.4%) vertebrates (Figure 1).

We found substantial disparities between the species defined as invasive by the national registries and the total set of species in state level invasives‐related policies. There was overlap in species named in state policies and included in the US‐RIIS or NAS databases, albeit with a smaller subset of species (*n* = 630 compared to 1862 species in the state database; see Table 2). The largest difference with the subset listed in the US‐RIIS or NAS databases was a proportionally larger number of plants and much smaller proportion of vertebrates. The total number of species named in both state policies and in the federal databases ranged from 30 to 187 by state (Table 2), with plants accounting for 57.1% (*n* = 360), invertebrates accounting for 28.4% (*n* = 179), and vertebrates accounting for 14.4% (*n* = 91). Among the subset of US‐RIIS/NAS named species, 143 (48.3%), 86 (29.1%), and 28 (9.5%) plant, invertebrate, and vertebrate species, respectively, were listed in only one policy chapter across all states. Within each state, the subset of US‐RIIS/NAS listed plants named in at least one policy ranged from 11 (Rhode Island) to 115 (New York), invertebrates ranged from 1 (Kentucky and Pennsylvania) to 69 (Mississippi), and vertebrates ranged from 0 (Pennsylvania) to 30 (Virginia; Appendix S1: Table S2).

**TABLE 2:** The total and unique (without counting the same species multiple times in separate policy chapters) records of listed species in invasive species related policy in 21 eastern US states that appear in US Register of Invasives and Introduced Species or the USGS Nonindigenous Aquatic Species databases.

State	Total species listed in chapters	Unique invasives listed in chapters	No. neighbors	No. unique species in neighborhood	No. in common	Among‐neighbor overlap (%)	Regional overlap (%)
Alabama	170	82	4	294	56	68.3	19
Connecticut	163	112	3	252	81	72.3	32.1
Delaware	157	81	3	164	48	59.3	29.3
Florida	472	188	2	104	46	24.5	44.2
Georgia	126	47	5	320	41	87.2	12.8
Kentucky	108	33	3	144	28	84.8	19.4
Massachusetts	164	97	5	286	67	69.1	23.4
Maryland	202	75	4	186	53	70.7	28.5
Maine	134	70	1	124	53	75.7	42.7
Mississippi	343	120	4	117	47	39.2	40.2
North Carolina	207	94	4	175	55	58.5	31.4
New Hampshire	510	124	3	140	77	62.1	55
New Jersey	215	72	3	252	39	54.2	15.5
New York	335	180	6	258	92	51.1	35.7
Pennsylvania	150	65	5	294	52	80	17.7
Rhode Island	184	49	3	269	35	71.4	13
South Carolina	195	79	2	118	46	58.2	39
Tennessee	102	61	6	263	47	77	17.9
Virginia	340	85	5	209	64	75.3	30.6
Vermont	81	30	3	280	27	90	9.6
West Virginia	92	57	4	169	37	64.9	21.9

*Note*: Among‐neighbor overlap shows the percent of invasive species in states that occur in at least one neighboring state. Regional overlap represents the percent of species that the focal state has in common with the entire neighborhood of adjacent states. These metrics show different facets of policy consistency among states.

**FIGURE 2:**
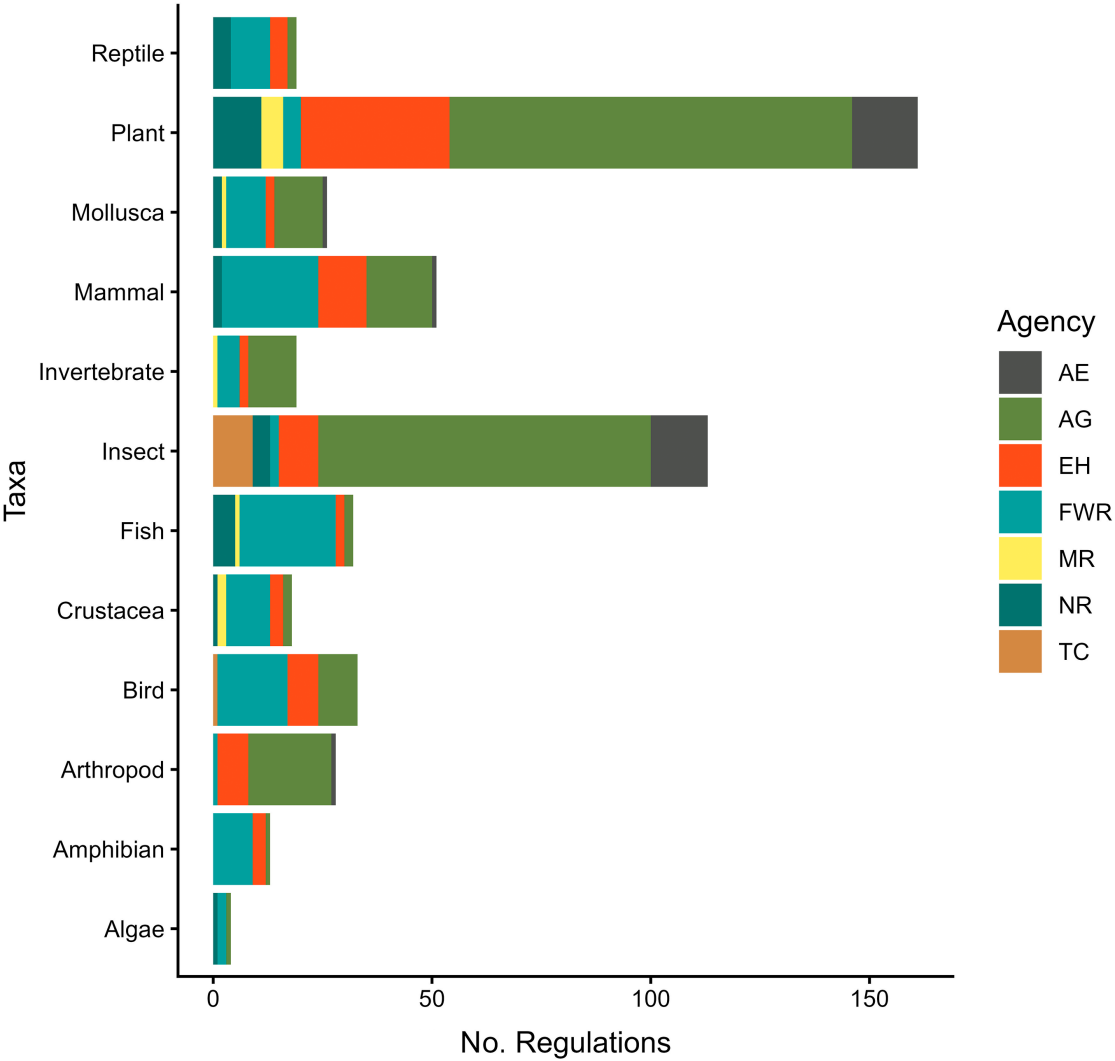
The distribution of regulations across different agencies for broad taxonomic categories of species and taxa mentioned in policy chapters for the 21 eastern US states in our database. These broadly fell across regulatory agencies related to agricultural, plant, or livestock industries (AG); wildlife, fisheries, and recreation (FWR); forestry, mining, and natural resources (NR); marine resources (MR); public and environmental health (EH); transportation, utilities, and commerce (TC); and administration, education, specific locations, and all other types (AE). The taxa identified could include a specific species and the broad category the species belongs to, as well as another taxa impacted by the invader (e.g., spotted lanternfly, *Lycorma delicatula*, may be specifically named, but also described as an invasive *insect* impacting local *plants* in the policy chapter).

The policy chapters included 638 regulations and 479 statutes. Regulatory agency types included in our policy chapters belonged to those related to agriculture, plant, or livestock industries (AG, *n* = 248); wildlife, fisheries, and recreation (FWR, *n* = 84); forestry, mining, and natural resources (NR, *n* = 63); marine resources (MR, *n* = 20); public and environmental health (EH, *n* = 151); transportation, utilities, and commerce (TC, *n* = 29); and admin, education, specific locations, and all other types (AE, *n* = 43; Figure 2, Appendix S1: Figure S1). Chapters related to aquatic species, habitat, and introduction pathways were only 17.1% of the total compared to 55.4% that referenced only terrestrial species, habitats, and pathways (*n* = 619), or the 27.5% including both (*n* = 307). We found that policy not only referenced specific species, but also broad taxonomic categories, often related to the broader taxonomic group of the invasive species or those local flora and fauna impacted by the invader. However, this was inconsistent across chapters (Figure 2, Appendix S1: Figure S1).

### Policy chapter consistency

**FIGURE 3:**
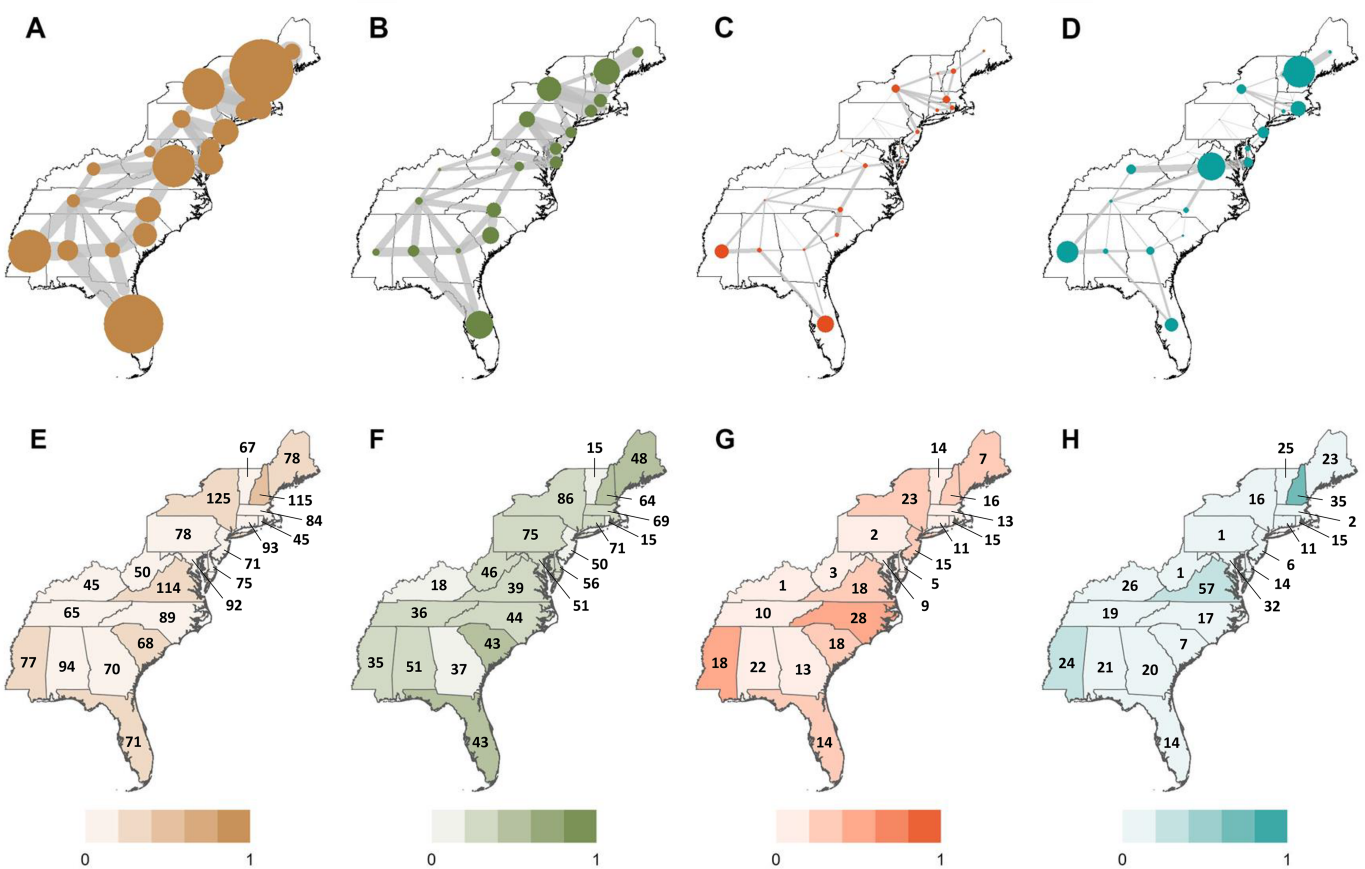
The relative number of policies that reference specific taxonomic groups (A–D) and relative consistency in policy adoption among states as measured by regional overlap of policies naming species (E–H) where numbers represent the number of species the state has in common with at least one state in the neighborhood. Columns represent *all taxa* (A, E), *plants* (B, F), *invertebrates* (C, G), and *vertebrates* (D, H). The number of species listed in policies of each state is represented by the relative size of the state centroid node. The width of the edges among these nodes represents the number of species in common among adjacent state pairs. For example, *all taxa* (A) values comprise the total sum of plants, invertebrates, and vertebrates listed in each state. Invertebrates are drastically underrepresented in policy relative to plant species in most states, while a few states disproportionately list many vertebrate species relative to their neighbors. This lack of consistency among policy in adjacent state neighborhoods shows via the intensity of the color among states. However, regional overlap may be high for a focal state if most states in the neighborhood list few species.

Considering all species named in each state's policy codes, the among‐neighbor consistency median and mean values were 53.8% and 52.2% (±16.3% SD) respectively (Table 1), meaning that about half of the listed species taxon in each focal state's policies are also listed in at least one neighboring state's policies. The range of among‐neighbor overlap was 20.6% (Florida) at the lowest to 80.6% (Vermont) at the highest, demonstrating high variability in the proportion of taxa listed in policies among each state and its neighbors. Regional overlap of policy chapters which measures a state's overlap with invasive species policies in all neighboring states, however, showed lower policy chapter consistency with median and mean values of 18.4% and 19.9% (±10.5% SD) respectively (Figure 3). For instance, Tennessee had the lowest overlap (8.8%) with species listed in all neighboring states while half of the species listed in New Hampshire (50.7%) were also found in the policies of neighboring states of Maine, Vermont, and Massachusetts.

Consistency metrics of among‐neighbor overlap and regional overlap varied considerably among taxa. Consistency as measured via among‐neighbor overlap was highest for plants with median and mean of 70.8% and 67.5% (±14.3 SD), respectively. Among‐neighbor overlap consistency for invertebrates was lower than for plant taxa, with median and mean values of 50% and 52% (±20.9 SD), respectively. Vertebrate taxa consistency was lowest, with median and mean of 33.3% and 36.8% (±23.5% SD), respectively (Appendix S1: Table S1; Figure 3).

Regional overlap of policy chapters for plants among neighboring states had median and mean values of 28.1% and 28.2% (±12.6% SD). Regional overlap of policy chapters for invertebrates had median and mean values of 17.9% and 19.2% (±12.5% SD), respectively. The regional overlap of vertebrate taxa in policy chapters had median and mean values of 8.2% and 14.1%, respectively (±16.6% SD; Appendix S1: Table S1; Figure 3).

For the subset of species listed as invasive in US‐RIIS/NAS, consistency of among‐neighbor overlap increased relative to the full dataset but trends were similar (Appendix S1: Table S2; Figure S2). This is potentially due to fewer species considered through the filtering lens of the US‐RIIS and NAS databases (Appendix S1: Figure S3). It could also be attributed to states' use of such lists to inform policy. For an expanded description of the consistency metrics among taxa and their range among states, please refer to Appendix S1.

### Pairwise distance and common species correlation

**FIGURE 4:**
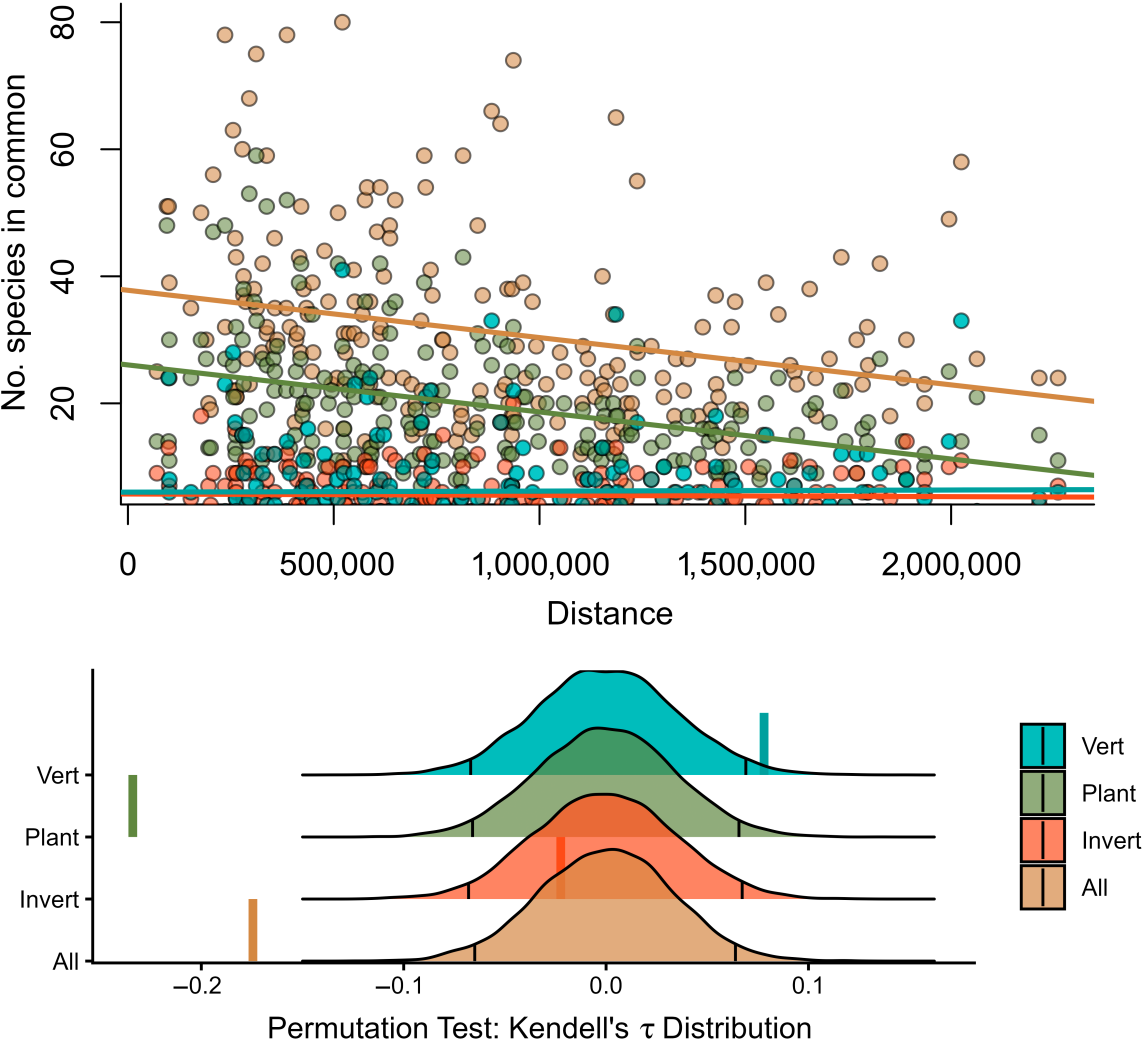
Correlation among pairwise distances between state centroids and the number of species a state has in common within its policy chapters. As distance increases, the number of species in common decreases. This trend is significant (*z* = −5.371; *p* = <0.001; τ = −0.1932776) for all taxa combined, but only for plants (*z* = −7.7268; *p* = <0.001; τ = −0.2574165) when separated. Distribution of test statistic values from a permutation test shows that this relationship holds when all taxa are lumped together (*z* = −5.246; *p* = <0.001; τ = −0.1745014), but is only significant for plants (*z* = −6.985; *p* = <0.001; τ = −0.2338575) when taxonomic groups are considered individually. Vertical colored lines represent the actual value of the Kendall's τ test statistic and are colored coded to the taxa in the distribution. Vertical black lines within the distribution curve show the 2.5% and 97.5% quantiles needed to find significance.

The correlation between pairwise distance among states and the number of species in common in policy chapters for all taxa was significant and negative (*z* = −5.371; *p* = <0.001; τ = −0.1932776). The longer the distance between state centroids, the fewer the number of named species in common among the state pairs (Figure 4). When different taxa were considered, only plants showed a significant (negative) correlation between the distance and the number of species in common among chapter policies (*z* = −7.7268; *p* = <0.001; τ = −0.2574165). The invertebrate (*z* = −0.60207; *p* = 0.5471; τ = −0.02056939) and vertebrate (*z* = 1.3511; *p* = 0.1767; τ = 0.04591663) taxa both showed no significant relationship among species listed in policy chapters and the pairwise distance of states. Permutation tests (*n* = 9999) showed these findings are robust, with Kendall's τ statistic for all taxa and plants‐only falling outside the 2.5% and 97.5% quantiles of a two‐tailed test for significance (Figure 4). The results for the subset of the dataset that includes only species found in US‐RIIS and NAS databases generally mirrored those of the full dataset (Appendix S1: Figure S4).

## CASE STUDIES

**FIGURE 5:**
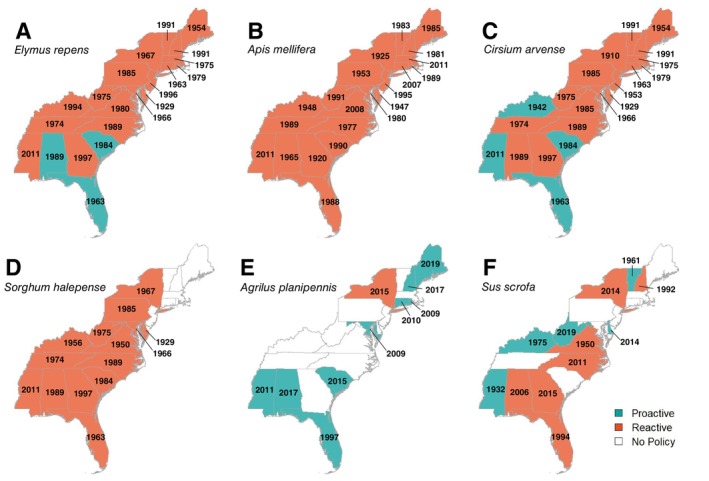
The policy landscape for the six case study species *Elymus repens* (A), *Apis mellifera* (B), *Cirsium arvense* (C), *Sorghum halepense* (D), *Agrilus planipennis* (E), and *Sus scrofa* (F). These species either had full coverage across the database (A–C) or were illustrative of the current policy limitations that may put regional management of invasives at risk to limited or late adoption of policy. Numbers in each state represent the first year a policy was enacted in that state referring to the species, while colors represent whether a species was *proactively* included in that policy before it was described to have occurred in the state (blue), if it was *reactively* included in policy after it was found to have occurred in the state (red), or if no policy was found there is no year included (white). Occurrence data was sourced from gbif.org (GBIF, 2024).

Here, we highlight six species from our database to illustrate cases of both policy cohesion and inconsistencies among states and evaluate the proactivity of state policies. We selected three of the four species named in all states (quackgrass: *E. repens*, western honeybee: *Ap. mellifera*, and creeping thistle: *C. arvense*—we omitted the fourth, dodder, as it was named at the genus level; Figure 5A–C) as examples of cohesive policy attention, though the number of states that have been proactive in regulating these species is worryingly small. We also identified an emblematic invasive species from each broad taxonomic category to explore in depth: Johnsongrass (*Sorghum halepense*) for plants, emerald ash borer (*Agrilus planipennis*) for invertebrates, and feral pigs (*Sus scrofa*) for vertebrates (Figure 5D–F). These six species demonstrate the breadth and depth of policy (in)consistency, coordination, and implementation.

### Complete coverage cases

Two plants and one invertebrate were named at the species level in policies in all 21 states. Of these three species, there is evidence of proactive policies for two, given that states enacted policies before they were detected in‐state (Figure 5A,C). Three of 21 states had proactive policies addressing quackgrass (*E. repens*; 14.3%), and four states had proactive policies addressing creeping thistle (*C. arvense*; 19%). Occurrence records of the western honeybee (*Ap. mellifera*) predate relevant policies in all states. These species are all non‐native to the United States, but the two plant species are generally considered problematic, while the non‐native honeybee is generally valued for pollination and honey production. This dichotomy is evident when we examine how states regulate each species. Policies that address quackgrass and creeping thistle focus on control and management, while policies on honeybees focus on protection. The creeping thistle is considered one of the more troublesome weeds in its invasive range (Tiley, 2010) and is one of the most listed noxious weeds across the United States (Skinner et al., 2000).

In contrast, federal legislation was initially designed to protect the honeybee from disease‐causing pathogens (e.g., “The Honeybee Act” of 1922). More recently, state and federal policies have broadened in scope to prevent and eradicate undesirable subspecies of non‐native *Apis* species (Marcelino et al., 2022). While the importation of new bees is regulated, there is no federal legislation to regulate *Ap. mellifera* already present within national borders. Instead, there is a patchwork of state regulations governing interstate movement of honeybees. The lack of consistency can increase the risk of spread of introduced pathogens, pests, and undesirable subspecies (Marcelino et al., 2022). These threats pose a risk to the US agricultural sector, which is largely dependent on western honeybees for insect‐pollinated crops (Jordan et al., 2021). Calderone (2012) estimated the contributions of honeybees to be worth over US$11.5 billion annually. However, recent work has shown that agricultural output is limited by a lack of pollinators (Reilly et al., 2020). Western honeybees may negatively impact native pollinator abundance and total agricultural output (Angelella et al., 2021), and these effects contribute to poorer human health outcomes (Smith et al., 2022). With the rapid human‐mediated spread of honeybees and their associated pests and pathogens, the public is more aware of potential negative impacts, leading to proposed actions to mitigate these threats. These efforts include a certification process and coordination of interstate movement using existing federal legislation as a template to reduce inconsistencies among states (Mailander & Grant, 2019; Marcelino et al., 2022).

### Johnsongrass

Johnsongrass occurs in all the states we examined except Maine (NRCS, 2025), and five states (all other states in New England) have detected presence of the invader but have no relevant policies. The remaining states have policies that refer to the species; of those with policies, all were reactive (Figure 5D). This may be due to the early establishment of Johnsongrass, which was likely unintentionally introduced in South Carolina circa 1830 as an agricultural seed contaminant (McWhorter, 1971). The species is known to be one of the most noxious weeds in mild climates across the globe, and its range is likely to expand polewards with climate change (Barney & DiTomaso, 2011). States that have no policies are located in the northern extent of the states in our database, leaving them in a likely path of invasion (Figure 5D). These states are either currently bordered by states where Johnsongrass is present (Maine) or have documented isolated occurrences (e.g., Massachusetts; GBIF, 2024). Proactive coordinated policy could help prevent negative impacts to agriculture, a sector that is of regional, political, and social importance. Johnsongrass is also likely to impact ecological resources in the region, such as limited meadow and grassland resources (e.g., Montague Sandplains, MA) that support rare and threatened species. Preventative measures and rapid response are likely important to curtailing this species as it is rhizomatous and has widespread herbicide resistance (Klein & Smith, 2021).

### Emerald ash borer

Emerald ash borer (EAB) is a relatively new invasive species in the United States, having been introduced sometime in the 1990s and first documented in Michigan in 2002 (Sun et al., 2024). EAB has been detected in all but two states in the database (Florida and Mississippi; https://www.emeraldashborer.info accessed July 30, 2025). Yet more states adopted policy proactively compared to the other examples (Figure 5E), likely due to the acknowledged speed and severity of impacts observed in invaded ranges. There are 10 states (47.6%) that have policy chapters that reference emerald ash borer; 9 of these were proactive chapters and 1 was reactive (Figure 5). However, the other half of the states examined have yet to adopt policy naming EAB directly, even though there have been detections of EAB in all of these states and federal quarantine policy occurred during outbreaks therein. This may be due to the speed of spread in high‐risk areas after initial detection in the United States (Sun et al., 2024). This type of invasion process makes it hard for legislators to react with appropriate speed compared to slower dispersers, such as Asian long horned beetles (*Anoplophora glabripennis*). While Asian long horned beetles are destructive to maple trees, they are slower to disperse, allowing quarantine and other regulatory efforts to be more effective after detection occurs in new locations (e.g., Coyle et al., 2021).

EAB has been one of the most costly and ecologically destructive forest insect pests introduced in North America, in that it has endangered native ash tree species (*Fraxinus* spp.) from forests in EAB's invasive range (Aukema et al., 2010; Duan et al., 2018; Herms & McCullough, 2014; Kovacs et al., 2010; Sun et al., 2024). EAB spread so quickly that in less than 20 years it was removed from domestic quarantine regulations by the United States Department of Agriculture (USDA) in favor of mitigation and control support (USDA, 2020). EAB can also negatively affect human wellbeing, for example reducing infant birth weight and increased lower‐respiratory tract infections in invaded areas (Donovan et al., 2013; Jones, 2018). Some regions proactively prepared to act collaboratively, given the known likely expansion and high risk to forests within their borders.

Cooperative arrangements add value to policy, increasing effectiveness across governmental and geographical scales. For example, the Ash Protection Collaboration Across Wabanakik (https://umaine.edu/apcaw) is a cooperative effort among federal, state, and tribal governments along with nongovernmental organizations to respond to EAB in northern New England and bordering Canadian and tribal lands. This cooperative is using research and outreach to maintain ash ecosystems for the Indigenous peoples of the region. Outreach can, but does not always, lead to private stakeholder action (Barnes et al., 2021), and can also help motivate policymakers (Alexander et al., 2020; Miller et al., 2017) to prioritize invasive species policy.

### Feral pigs

In the United States, feral pigs have become an increasingly problematic invasive pest, with their highest density in the southern states but with suitable habitat throughout North America and populations in some northeastern states (McClure et al., 2015). In our database, of the 12 states that have policy chapters that reference feral pigs, 5 were proactive and 7 were reactive (Figure 5F); the remaining 9 states did not have identified policy chapters for feral pigs. Of these nine without policies, at least three have feral pigs present and most are adjacent to invaded states (GBIF, 2024). Feral pigs were estimated to cause the US$1.5 billion dollars in damages and control costs in 2007 (Pimentel, 2007), but this figure is likely an underestimation because the density and distribution of feral pigs has expanded since 2007 (Lewis et al., 2019). Feral pigs are also known to spread diseases to wildlife, livestock, and humans (Miller et al., 2017) and cause damage to sensitive habitats and agriculture alike (Strickland et al., 2020). Yet a patchwork of policies and regulatory agencies across (and even within states) has hindered coordination for feral pig management (Figure 5F), particularly considering almost half of the states do not have a policy explicitly naming *S. scrofa*. Some states also have more than one agency tasked with managing feral pigs, which can represent competing interests such as maintaining pigs for hunting or encouraging their removal to protect agricultural interests (Centner & Shuman, 2015; Wildlife Society, 2012). Some states encourage the hunting of feral pigs, while others have outlawed the practice in an attempt to reduce interest in transporting the species for sport (e.g., New York; Centner & Shuman, 2015). Not all states, however, have outlawed the transport of feral pigs within their borders, and this lack of regulation correlates with their invasive presence (Smith, Strickland, et al., 2023). Prohibitive regulations, such as banning private landowners from profiting from feral pig hunting opportunities, also appeared to reduce the number of pigs spread by reducing incentive to illegally release the animals (Smith, Strickland, et al., 2023). However, such actions may not represent a reasonable policy in locations such as Florida where large feral pig populations require a targeted, responsive management approach.

While no national policy is in place, there has been an increase in requests for federal aid and action (Smith, Tomecek, & McKee, 2023). The increase in stakeholder requests, coupled with increased negative media attention, has garnered increased legislative support at the federal level (Miller et al., 2017), and public response has, in part, helped to secure funding for the USDA to have its Natural Resources Conservation Service (NRCS) and Animal and Plant Health and Inspection Service (APHIS) jointly implement the Feral Swine Eradication and Control Pilot Program (FSCP). This pilot project received US$75 million to explore control and restoration methods across several states (Smith, Tomecek, & McKee, 2023). Successful preliminary implementation of efforts across counties in 13 states (six of which were included in our study) involved the cooperation of 59 agencies and partners directly involved in FSCP projects, with 68 more providing additional support (Smith, Tomecek, & McKee, 2023). Although this federal and state investment is likely necessary to conduct the large‐scale control needed for feral pig control (Lewis et al., 2019; Strickland et al., 2020), it contrasts with public opinion that suggests that those responsible for the release of feral pigs and individual landowners should be the main parties responsible for the financial burden of control efforts (TuckerWilliams et al., 2024).

**FIGURE 6:**
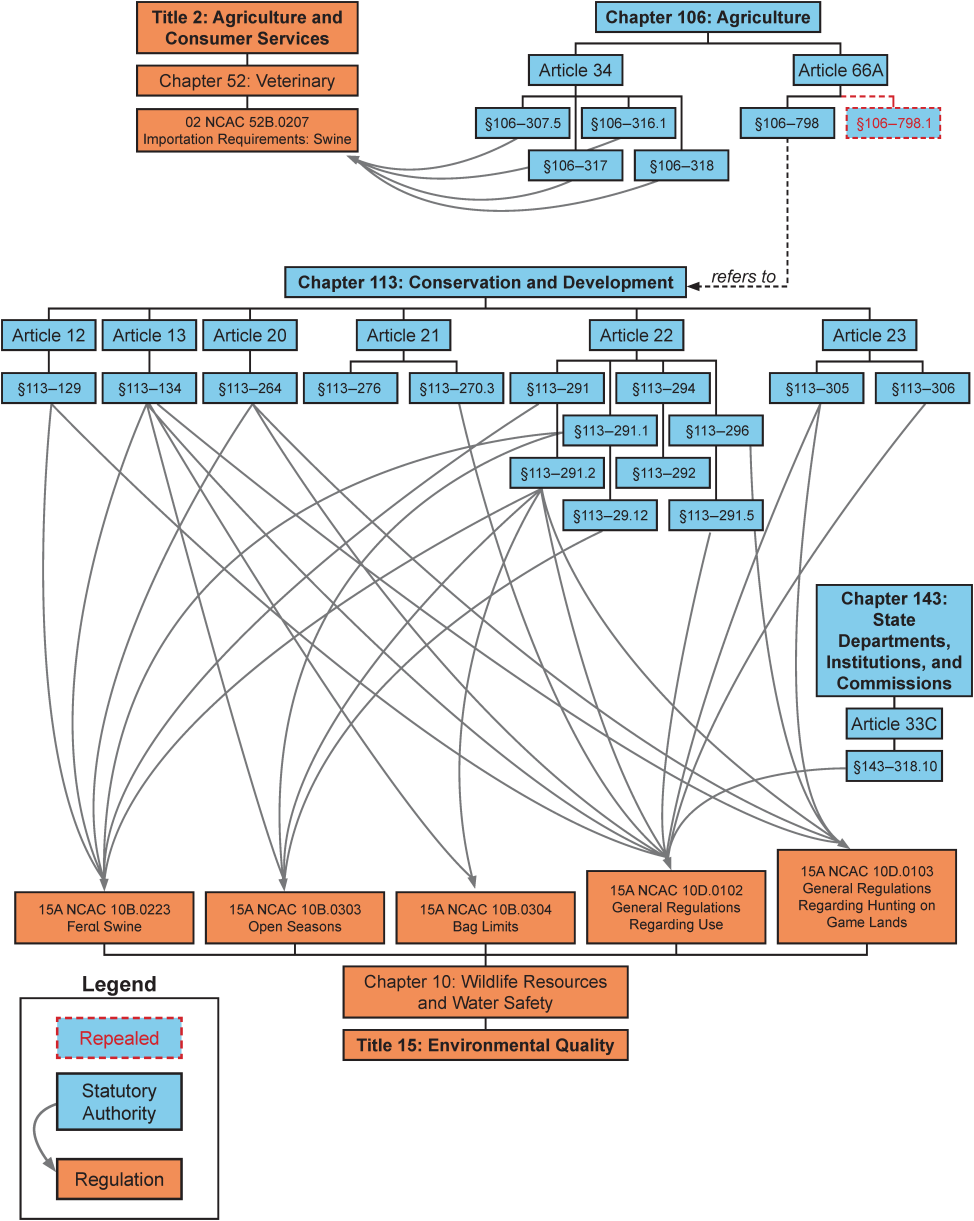
Diagram of the feral pig (*Sus scrofa*) policies in North Carolina and the pathway of interactions between statutes and regulation. This diagram shows the complexity of the policy landscape within a single state. However, this already complex network is only illustrative of those policies highlighted by the North Carolina Feral Swine Task Force and Wildlife Resources Commission (North Carolina Wildlife Resources Commission, 2024) and not the complete set of all policies that pertain to feral pigs in North Carolina.

Policy to address feral pigs at the state level is exemplar of the complexities of intertwined legislation and regulations, and of the potential for significant variability in foci, the nature and strength of management interventions, and variable responsibility among agencies and other actors across states. Figure 6 illustrates the web of policies in North Carolina (NC) as an example (note: the authors are not criticizing NC in this evaluation; quite the opposite: the transparency in agency documents on regulations pertaining to feral pigs is applauded; e.g., North Carolina Wildlife Resources Commission, 2024). In NC, feral swine are defined as any free‐ranging member of the species *S. scrofa*, regardless of their lineage (General Statute 113.129 (5c)). They are explicitly classified as “nongame, wild animals” and statutory authority over their harvest is given to the Wildlife Resources Commission (G.S. 113–129 (11d), G.S. 113–129 (15), G.S. 113–291.2 (a)). Reflecting a harvesting‐based eradication and control strategy, there are no bag limits and relatively few rules around when and how feral hogs are hunted and trapped (15A NCAC 10B .0223; 15A NCAC 10D .0103 (k); 15A NCAC 10B .0303 (b); North Carolina Wildlife Resources Commission, 2024). This strategy contrasts with that employed in other states, such as Kentucky, where hunting and trapping are banned or heavily regulated to disincentivize the illegal release and spread of wild pigs (Kentucky Department of Fish and Wildlife Resources, 2025). In North Carolina, the Department of Agriculture also plays key roles, regulating the importation and movement of hogs (02 NCAC 52B .0207) and in managing feral swine (North Carolina Department of Agriculture and Consumer Services, 2025). This illustrates the dynamic nature of the feral pig invasion and the resulting response that has resulted in a patchwork of codified and uncodified policies, which can lack clear pathways to application and management; further such outcomes are often idiosyncratic to each state (Smith, 2020).

## DISCUSSION

There is a major need for consistency in invasive species policies to enhance coordination and increase neighboring state governments' abilities to collaboratively prevent and prepare for future threats (Beaury et al., 2021; Lakoba et al., 2020; Reed, Cathey, et al., 2023). However, we found a general lack of consistency among state policies for invasive species across taxonomic groups. Our findings are consistent with previous studies that show limited policy coherence among neighboring states for invasive plants and noxious weeds (e.g., Beaury et al., 2021; Lakoba et al., 2020). The number of plant species named in policies is relatively high compared to other taxonomic groups we evaluated, and policies listing invasive plants have higher among‐neighbor and neighborhood overlaps compared to policies pertaining to invertebrate or vertebrate species (Appendix S1: Table S1). The greater overlap of listed plants may reflect the historical regulation of agricultural or noxious weeds in addition to recent additions of new invasive plant‐related policies among neighboring states. For example, there are existing policies to regulate invasives in the horticulture trade, which often extends across state borders. Yet, inconsistent regulations may cause confusion and facilitate unintended spread (Hulme et al., 2018).

The relative paucity of policies attending to invertebrates and vertebrates may simply reflect the limited degree of coordination across all taxa. Similarly, Foster et al. (2024) found that there is significant spatiotemporal variation and inconsistency in investment for both proactive and reactive measures against invasive species across the states. Such inconsistencies may lead to inaccurate estimates of the economic cost of invasive species, reducing the ability to effectively plan and coordinate management and prevention efforts (Vaissière et al., 2022). Developing policies that increase coordination, transparency, and the tracking of costs may help states to more effectively plan and conduct invasive species prevention and management (Foster et al., 2024; Hulme et al., 2024).

The lack of attention to invertebrates and vertebrates in our data (19% and 14% neighborhood overlap respectively) does not reflect the national or global risks and costs incurred by both taxa (Bacher et al., 2023; Fantle‐Lepczyk et al., 2022). State policies may also not accurately reflect the efforts that resource agencies, regulators, and industries contribute to invasive species management. For example, in Virginia, the Virginia Department of Forestry is empowered to identify and facilitate the management of insect infestations without codified policy identifying any specific species. Many other states also have invasive species and agricultural pest related services that are not fully reflected in statutes and regulations. This approach has benefits, such as providing regulators flexibility in responding to new problems (i.e., a permitted list approach; Patoka et al., 2018; Reed, Cathey, et al., 2023). However, the disconnect between policy and on‐the‐ground actions poses a problem insofar as the informality can act as a barrier to effective communication and compliance (Reed, Cathey, et al., 2023). Stakeholders (e.g., import traders, suppliers, and NGOs) may not know where to look to find appropriate information. Further, different agencies may be more prone to unnecessarily duplicate efforts or unwittingly miss opportunities for collaborative management on common goals in neighboring states.

Species that are more mobile are not as highly constrained to existing networks and economic pathways, unlike invasive plants in the horticulture industry. As a result, such mobile species will be harder to manage using policies and prohibited lists, particularly after establishment. Managing some animals may also be easier to address through policy than others due to social acceptance. The most cost‐effective, efficient, and impactful control methods may be socially unacceptable, resulting in a socioecological disparity (Beever et al., 2018; Roberts et al., 2018; Wald et al., 2013). Thus, codified policy may be limited to specific methods or agencies and omit species altogether, reducing the efficacy of certain policies. If social acceptance is garnered, it may be after the spread and establishment of the invasive species. Yet regulations adopted post‐invasion may be less effective, particularly for those that can move independently of trade pathways and human networks. Since codified policy often takes years or decades to pass and enact, it is likely that proactive measures will have the best return on investment (Branco et al., 2021; Cuthbert et al., 2022; Funk et al., 2014).

Climate change is likely to reduce the barriers to spread and establishment for non‐native species to become invasive (Bradley et al., 2023; Finch et al., 2021; Skendžić et al., 2021). Thus, the threat posed to both economic and ecological systems urgently requires more attention from policymakers (Bradley et al., 2021, 2023; Lovett et al., 2016). For example, forest insect pests have been most concentrated in the northeastern United States (Liebhold et al., 2013) including the spongy moth, *Lymantria dispar*, the most costly invasive species in the region (Fantle‐Lepczyk et al., 2022). However, the states with the highest number of species listed are Florida and Mississippi. Forest insect pests and pathogens are also the most costly invaders for the forestry sector across the entire eastern United States, particularly in the southeastern states, potentially helping explain the disproportionate number of listings in Florida and Mississippi (Fantle‐Lepczyk et al., 2022). Yet, in the eastern US, five of the six most costly invasive species were invertebrates and the last was *S. scrofa*, a mammal (see above; Fantle‐Lepczyk et al., 2022). It is possible that comparatively more invertebrate related policies would be found in other states not included in this database, as significant damages from insects have been recorded in both the midwestern and western United States (Fantle‐Lepczyk et al., 2022), but this would not account for the entire underrepresentation of invertebrates in policies found in our database, particularly given the impact they have within the region. Many invasive species are already present in many states, yet unregulated, and this increases the threats among neighboring states (Aukema et al., 2010; Paini et al., 2010). Thus, even proactive states can be susceptible to invasion from “weak link” neighboring states that do not adopt similar policies (Keller et al., 2011; Klizentyte et al., 2021; Paini et al., 2010).

Spatial inconsistencies among states are likely to complement temporal inconsistencies at the federal level as vacillations in policy occur during changes in administration. This could be largely resolved with legislation focusing on invasive species policy, but in the absence therein, having state‐level cooperation that can operate smoothly as federal administrations change priorities would be (next) best practice. To complement this, some federal regulation or law that could help “pull‐up” less proactive states would provide some insurance for proactive regional actors. This could extend internationally, as species do not stop at state or international borders. Coordination will be important among northern states and Canadian provinces to plan as changing climates facilitate invasive spread northward.

While the development of coordinated efforts is important to successfully prevent, manage, and mitigate invasive species (Hulme, 2016), it is clear from this and other work that the current legislative policy tools fall short (Hulme, 2021b), and such inadequacies leave many states that invest heavily at the risk of threats from neighbors that do not have similar policies and investment (Figures 3 and 4). There has been increased investment and federal programs within the United States (e.g., Noxious Weed control and Eradication Act of 2004 and FSCP) to help fund the eradication of invasive species. However, the lack of consistency among state policies may be reflective of a lack of national policy direction on how to establish, when, and what an invasive species is (Klizentyte et al., 2021). Some risk assessments for noxious weeds require the species to be present before being listed as invasive, a detrimental barrier to proactive and coordinated policy and management action (Beaury et al., 2021). There may be ways to harness or copy existing policy and infrastructure to help coordinate management action through federal funding mechanisms that tie into regional invasive priorities and needs akin to the competitive and non‐competitive State Wildlife Grants program administered by the U.S. Fish and Wildlife Service. We note that these funds should complement, not be reallocated from, current conservation efforts.

Investment in expeditiously communicating findings among policymakers, practitioners, and researchers has increased through the adoption of federal policies such as the Early Detection Rapid Response framework (EDRR; Reaser, Burgiel, et al., 2020; Reaser, Simpson, et al., 2020). However, there remain many legislative and regulatory gaps in the ability to conduct such policies at the federal level (Burgos‐Rodríguez & Burgiel, 2020), leaving them open to interpretation and cessation through executive actions. From the practitioners' perspective, research may not be meeting needs even though it is accessible (Matzek et al., 2015), but there are opportunities to use existing research and outreach structures such as the extension model used at US Land Grant institutions (Barney et al., 2019). The identification of research priorities and the transfer of generated knowledge could also be improved through frameworks such as translational invasion ecology. These frameworks explicitly take into account the ecological, sociological, economic, and political contexts (Morelli et al., 2021). Incorporating diverse stakeholders and quantitative tools such as using simulations, climate change projections, connectivity assessments, and species distribution maps that take into account the socioeconomic components may be able to more holistically evaluate and act upon the potential threats (Beaury et al., 2020; Lakoba et al., 2020; Reed, Schenk, et al., 2023).

Proactive policy informed by quantitative tools may be increasingly important for coordinated prevention and rapid response as climate change allows species to expand into new locations or relax the socioecological limiting conditions on established non‐native species that may facilitate a transition from being largely benign to invasive (Bradley et al., 2024; O'Uhuru et al., 2024; Reaser, Burgiel, et al., 2020; Spear et al., 2021). For example, O'Uhuru et al. (2024) have predicted increased climate suitability for a suite of non‐native species in the New England region and highlighted 18 that could be the highest priority for proactive prevention and rapid response and eradication. Within our database, we found that only four of these are currently found in policies in at least one state defined in their New England study region. A fifth is in our database but outside of their study region. The remaining 13 species identified by O'Uhuru et al. (2024) did not occur in policies within the 21 states in our database, although some states do have closely related species present in policies in the database. Nine of those 13 species were identified as being likely to have negative social and economic impacts in addition to either moderate or severe ecological impacts (O'Uhuru et al., 2024). Understanding these potential threats may help provide the information to implement climate‐smart invasive species policy across regions (Beaury et al., 2020; Bradley et al., 2023). Given that such proactive action is more cost‐effective than reactive responses and damage mitigation (Fantle‐Lepczyk et al., 2022; Foster et al., 2024), increased attention should be given to researching species likely to become problematic in the future, which can then be identified for coordinated policy responses. While this work has largely focused on non‐native plants, other taxa should not be ignored as both vertebrates (Courchamp et al., 2003; Spear et al., 2021) and invertebrates (Frank & Just, 2020) can act as “sleeper” species.

## CONCLUSION

Although many future invasions are preventable (Roy et al., 2023), lack of coherent and consistent policies across states and regions could continue to undermine prevention and rapid response efforts, even though such efforts are the most economically expedient course of action (Fantle‐Lepczyk et al., 2022; Keller et al., 2007; Roy et al., 2024). Existing frameworks (e.g., EDRR or One Biosecurity) could provide insight for moving forward to accomplish more integration and cooperation of invasive management through policy that reaches across borders (Hulme, 2021b; Quinn et al., 2013; Reaser, Burgiel, et al., 2020). The large variety of sectors and scale of impacts provides impetus for more urgent political and public attention to invasive species (Pyšek et al., 2020). While uniform policies can be detrimental if no flexibility or reasonable accounting for idiosyncratic conditions is provided (Albers et al., 2010), too much subdivision of responsibility can hinder cooperation necessary for collective action to achieve successful control efforts (Figures 2 and 6; Epanchin‐Niell et al., 2010). Our work suggests that the current scope of policies in the 21 eastern US states included in our database lacks consistency, particularly for invertebrate and vertebrate taxa. While our analyses are regionally focused, our results concur with previous work that examines plant‐based policies and regulations across the entire United States. Our findings could be representative of the policy landscape for non‐plant taxa across the United States, but more research would be needed to confirm. The incoherence in policy undermines the ability to respond to existing and emerging invasive threats. Even without a guiding regulatory framework from the federal government, opportunities exist to increase coordination at the state level. However, paralleling federal and state level reform to facilitate synergy of policy objectives could help promote coordination of responses to invasive species most effectively (Beaury et al., 2021; Klizentyte et al., 2021), and could simultaneously reduce counterproductive and disparate management actions while also encompassing increased support and coordination to manage invasive species (Roy et al., 2024).

## AUTHOR CONTRIBUTIONS

Joseph Drake, E. M. X. Reed, David Haak, Bryan Brown, Meryl C. Mims, Michael G. Sorice, Haldre Rogers, Scott Salom, Todd Schenk, and Jacob N. Barney conceived the ideas and designed the methodology for the paper. Joseph Drake conducted analyses for the paper. Joseph Drake led the writing of the manuscript. All authors contributed critically to the drafts and gave final approval for the publication.

## CONFLICT OF INTEREST STATEMENT

The authors declare no conflicts of interest.

## Supporting information


Appendix S1.

## Data Availability

Data (Haak & Drake, 2026) are available in the University Libraries, Virginia Tech repository at https://doi.org/10.7294/31930884.
