# Supplementary material for: State lines and species divides: Inconsistencies and opportunities in invasive species policies across the eastern United States
Source: Ecol Appl. 2026 Aug 2;36(5):e70287. doi: 10.1002/eap.70287 (PMC13430114; doi:10.1002/eap.70287)
Supplement: Supplementary file 1 — Appendix S1. [file EAP-36-e70287-s001.pdf]

Appendix S1

State lines and species divides: Inconsistencies and opportunities in invasive species policies across the eastern United States

Joseph Drake, E.M.X. Reed, David Haak, Bryan Brown, Meryl C. Mims, Michael G. Sorice, Haldre Rogers, Scott Salom, Todd Schenk, and Jacob N. Barney

Ecological Applications

Glossary

Glossary of terms related to invasive species policy. For more description of the structure of state policies relative to the data presented here, see Reed et al. 2023.

Chapter - generally smaller subsections of legal documents. Here more specifically, because code and regulations are not formatted similarly across states, chapter is used as a common policy unit across states.

Codified - a codified law/policy is one written or passed by a legislative body in a formal manner, often consolidating judicial rulings, case law, customary practices, or uncodified statutes. Uncodified law/policy is not relegated to a single formal document, code, or system, but through precedent contribute to case law, customary practices, etc..

Federal - referring to national (United States of America) level policy, regulations, and statutes.

List - a list of species that are of interest for specific purpose (e.g., noxious weed lists), which may or may not be codified and thus hard to compare or find.

Policy - the system or collection of guidelines which provide a structure for decision making to achieve particular desired outcomes.

Regulations - rules, usually written by administrative agencies, granted legal authority through statutes.

Sections - a form of subdivision of a legal document.

Statutes - laws that are enacted by a legislative body.

Permitted list - laws/policies that apply to all species except those named; previously referred to as “whitelists” and the opposite would be a “blacklist” law.

Reference

Reed, E.M., S. Cathey, C. Braswell, P. Agarwal, J.N. Barney, B.L. Brown, A. Heminger, A. Kianmehr, S. Salom, T. Schenk, and G. Sharma. 2023. The state of play in invasive species policy: Insights from invasive species laws and regulations in 21 US states. BioScience 73(10), pp. 738–747.

Expanded Narrative Results

Policy overview

To date, there are 1117 policy chapters in the 21 eastern U.S. states in our database; 411 new policies were added to the original 706 presented by Reed et al. (2023a). Of the full dataset, 448 policy chapters name at least one taxa at the genus or species level (hereafter referred to as species). The total number of species (counting species named in multiple policies) that occur in policy chapters within each state ranges from 81 (Vermont) to 510 (Florida). Within these policy chapters, there are 1682 unique species named across all states (Table 1, main text). Within each state the number of species listed ranges from 67 (Vermont) to 345 (Florida). The majority (1017; 60.5%) of species are only recorded in a single state (Figure 1, main text). Approximately 7% of listed species (119) are recorded in >5 states. Only four species are recorded in policy chapters across all 21 states in the database: western honey bee (*Apis mellifera*), creeping thistle (*Cirsium arvense*), couch grass (*Elymus repens*), and dodder (*Cuscuta spp*).

When broken down by taxonomic group, approximately 35.3% (594) of all listed species are plants, 19.9% (335) are invertebrates, and 44.8% (753) are vertebrates. The number of plants named in at least one policy within a state ranges from 20 (Rhode Island) to 161 (New York), invertebrates range from 2 (Kentucky) to 105 (Mississippi), and vertebrates ranged from 3 (Massachusetts) to 189 (Virginia; Appendix S1:Table S1). The number of species that is only listed in one policy chapter across all states is 241 (40.6%), 203 (60.6%), and 425 (56.4%) for plants, invertebrates, and vertebrates respectively (Figure 1, main text). The median number for all policy chapters found for each state was 49 (range: 21 to 149 chapters), yet when considering only chapters that specifically name species, the median dropped to 21 chapters (range: 9 to 46 chapters).

When we only consider species both named in state policies and named by US-RIIS or NAS databases, the general patterns are similar if with a smaller subset of species (n=630; Table 2, main text). The largest change between the subset only listed in US-RIIS/NAS database was for an increase in the proportional contribution of plants to the overall dataset while the proportional contribution of vertebrates decreased substantially. Total number of species for this subset named in the policy database ranged from 30 to 187 (Table 2, main text). For US-RIIS/NAS named species, plants accounted for 57.1% (n=360), invertebrates accounted for 28.4% (n=179), and vertebrates accounted for 14.4% (n=91) of the species named in state policy chapters. This subset of US-RIIS/NAS named species has 143 (48.3%), 86 (29.1%), and 28 (9.5%), for plants, invertebrates, and vertebrates respectively listed in one policy chapter across all states. The subset of US-RIIS/NAS listed plants named in at least one policy within a state ranges from 11 (Rhode Island) to 115 (New York). The number of invertebrates named at least once in a state's policies range from 1 (Kentucky & Pennsylvania) to 69 (Mississippi). The number of vertebrates that were named at least once in state policies ranged from 0 (Pennsylvania) to 30 (Virginia; Appendix S1: Table S2).

Policy Chapter Consistency

Considering all species named in a state's policy codes, the among-neighbor consistency median and mean values were 53.8% and 52.2% (± 16.3% SD), respectively (Table 1, main text). The range of among-neighbor overlap was 20.6% (Florida) at the lowest and 80.6% (Vermont) at the highest, demonstrating high variability in the proportion of taxa listed in policies among a focal state and its neighbors. Regional overlap of policy chapters, however, shows lower policy chapter consistency with median and mean values of 18.4% and 19.9% (±

10.5% SD) respectively (Figure 3). The regional overlap ranged from a low of 8.8% (Tennessee) to a high of 50.7% (New Hampshire).

Consistency metrics of among-neighbor overlap and regional overlap varied considerably among taxa. Consistency as measured via among-neighbor overlap was highest for plants with median and mean of 70.8% and 67.5% (± 14.3 SD) respectively (Appendix S1: Table S1; Figure 3). These values ranged from a low of 26.9% (Florida) to a high of 92.5% (Georgia). Regional overlap of policy chapters among neighboring states had median and mean values of 28.1% and 28.2% (± 12.6% SD). Regional overlap ranged from 6.2% (Rhode Island) to 50% (Maine).

Among-neighbor and regional overlap consistency for invertebrates was lower than for plant taxa. Consistency measured by among-neighbor overlap had median and mean values of 50% and 52% (± 20.9 SD) respectively (Appendix S1: Table S1; Figure 3, main text). The among-neighbor overlap range was largest for any taxa examined, ranging from 13.7% (Florida) to 93.3% (Vermont). Regional overlap of policy chapters among neighboring states had median and mean values of 17.9% and 19.2% (± 12.5% SD) respectively. The range of regional overlap was from 2% (Pennsylvania and Kentucky had only 2 and 1 species in common with their neighbors respectively) to 41.9% (Mississippi). Notably, while Mississippi had a slightly higher proportional regional overlap, its total in common species with its neighborhood was 18. However, North Carolina had nearly as high a regional overlap with 41.8% but had 28 species in common with their neighborhood (Figure 3, main text).

Vertebrate taxa consistency, as measured by among-neighbor overlap, had a median and mean of 33.3% and 36.8% (± 23.5% SD), respectively (Appendix S1: Table S1; Figure 3, main text) and was the highest standard deviation of the defined taxonomic groups showing high variability among overlap with neighbors. The range was also large, from a low of 7.1% (New

Jersey) to 86.2% (Vermont).The regional overlap of vertebrate taxa in policy chapters had a median and mean values of 8.2% and 14.1% (± 16.6% SD), representing the highest divergence in the measures of central tendency among the defined taxa. The range of regional overlap varies from a low of 0.3% (West Virginia) to a high of 74.5% (New Hampshire). Massachusetts and Pennsylvania both have low regional overlap with 0.6% (2 species) and 0.5% (1 species) respectively.

For the subset of species listed as invasive in US-RIIS/NAS consistency of among neighbor overlap increased relative to the full dataset This is likely due to fewer species considered through the filtering lens of the US-RIIS and NAS databases. Consistency of plant among-neighbor overlap ranged from 32.3% to 95.8% and had median and mean values of 72.7 and 71.4% (± 14.5% SD) respectively; regional-overlap ranged from 5.7% to 57.1% and had median and mean values of 32.1% and 30.4% (±14.5% SD) respectively (Appendix S1: Table S2; Figure S2). Consistency of invertebrate among-neighbor overlap ranged from 12.5% to 100% and had median and mean values of 65% and 62.5% (± 25.0% SD) respectively; regional-overlap ranged from 1.8% to 54.2% and had median and mean values of 23.1% and 23.6% (± 14.4% SD) respectively (Appendix S1:Table S2; Figure S2). Consistency of vertebrate among-neighbor overlap ranged from 0% to 100% and had median and mean values of 60.0% and 56.9% (± 27.9% SD) respectively; regional-overlap ranged from 0% to 66.7% and had median and mean values of 22.9% and 23.6% (± 17.8% SD) respectively (Appendix S1: Table S2; Figures S1 & S2).

Pairwise distance and common species correlation

Correlation between pairwise distance among states and the number of species in common in policy chapters for all taxa show a significant negative correlation (z=-5.371; p=

<0.001; **τ**=-0.1932776). The longer the distance between state centroids, the fewer species in common are named at least once among the state pairs (Figure 4, main text). When different taxa are considered, only plants show a significant correlation among pairwise distances of state pairs and the number of species in common among chapter policies (z= -7.7268; p= <0.001; **τ**= -0.2574165). Both the defined groups of invertebrate (z= -0.60207; p= 0.5471; **τ**= -0.02056939) and vertebrate (z= 1.3511; p= 0.1767; **τ**= 0.04591663) taxa show no significant relationship among species listed in policy chapters and the pairwise distance of states. Permutation tests (n=9999) show these findings are robust with all taxa and plants only groups' only plants Kendall's **τ** statistic falling outside the 2.5% and 97.5% quantiles of a two-tailed test for significance (Figure 4, main text). The results of the subset of the dataset which includes only species found in US-RIIS and NAS databases generally mirrors those of the full dataset (Appendix S1: Figure S4). The correlation and permutation tests for pairwise distance among states and number of invasive species shows negative correlation for all taxa (z=-5.246; p= <0.001; **τ**=-0.1745014) and for plants only (z=-6.985; p= <0.001; **τ**=-0.2338575). There was limited evidence for a significant correlation for invertebrates (z=-0.647; p= 0.517; **τ**=-0.02240287). However, there was slight evidence for vertebrates (z=-2.2505; p= 0.02442; **τ**=0.07807211) to show a positive relationship, although the strength of that correlation is extremely low; permutation tests also show this to be just outside of 97.5% confidence bounds (Appendix S1: Figure S4).

Tables & Figures

Table S1. The total and unique (without counting the same species multiple times in separate policy chapters) records of listed species in invasive species related policy in 21 eastern U.S. states broken apart by major taxonomic groups and the calculated consistency metrics

Taxa	State	Total Species listed in Chapters	Unique Species listed in Chapters	Number Unique Species in Neighborhood	Number in Common	Among-Neighbor Overlap (%)	Regional Overlap (%)
Plants	Alabama	90	78	211	51	65.385	24.171
	Connecticut	99	98	212	71	72.449	33.491
	Delaware	97	91	166	56	61.538	33.735
	Florida	224	160	91	43	26.875	47.253
	Georgia	40	40	305	37	92.5	12.131
	Kentucky	28	22	111	18	81.818	16.216
	Massachusetts	102	97	245	69	71.134	28.163
	Maryland	99	72	178	51	70.833	28.652
	Maine	86	63	96	48	76.19	50
	Mississippi	56	51	106	35	68.627	33.019
	North Carolina	120	82	166	44	53.659	26.506
	New Hampshire	214	96	130	64	66.667	49.231
	New Jersey	90	69	261	50	72.464	19.157
	New York	196	161	251	86	53.416	34.263
	Pennsylvania	134	95	283	75	78.947	26.502
	Rhode Island	20	20	234	15	75	6.41
	South Carolina	141	102	100	43	42.157	43
	Tennessee	61	53	170	36	67.925	21.176
	Virginia	77	52	183	39	75	21.311
	Vermont	25	23	242	15	65.217	6.198
	West Virginia	71	58	160	46	79.31	28.75
Invertebrates	Alabama	37	35	203	22	62.857	10.837
	Connecticut	29	14	98	11	78.571	11.224
	Delaware	16	14	48	5	35.714	10.417
	Florida	140	102	48	14	13.725	29.167

	Georgia	21	19	160	13	68.421	8.125
	Kentucky	2	2	49	1	50	2.041
	Massachusetts	59	28	96	13	46.429	13.542
	Maryland	25	21	50	9	42.857	18
	Maine	21	21	32	7	33.333	21.875
	Mississippi	109	105	43	18	17.143	41.86
	North Carolina	42	38	67	28	73.684	41.791
	New Hampshire	41	32	50	16	50	32
	New Jersey	35	28	72	15	53.571	20.833
	New York	64	59	77	23	38.983	29.87
	Pennsylvania	5	3	101	2	66.667	1.98
	Rhode Island	37	32	84	15	46.875	17.857
	South Carolina	34	29	47	18	62.069	38.298
	Tennessee	16	14	162	10	71.429	6.173
	Virginia	38	29	69	18	62.069	26.087
	Vermont	20	15	96	14	93.333	14.583
	West Virginia	16	15	44	3	20	6.818
Vertebr-ates	Alabama	43	43	255	21	48.837	8.235
	Connecticut	35	34	187	11	32.353	5.882
	Delaware	44	42	171	14	33.333	8.187
	Florida	108	83	94	14	16.867	14.894
	Georgia	65	60	181	20	33.333	11.05
	Kentucky	78	77	204	26	33.766	12.745
	Massachusetts	3	3	309	2	66.667	0.647
	Maryland	78	78	233	32	41.026	13.734
	Maine	27	27	122	23	85.185	18.852
	Mississippi	178	141	64	24	17.021	37.5
	North Carolina	45	45	251	17	37.778	6.773

	New Hampshire	255	122	47	35	28.689	74.468
	New Jersey	90	85	114	6	7.059	5.263
	New York	75	68	261	16	23.529	6.13
	Pennsylvania	11	11	219	1	9.091	0.457
	Rhode Island	127	127	98	15	11.811	15.306
	South Carolina	20	20	102	7	35	6.863
	Tennessee	25	25	409	19	76	4.645
	Virginia	225	189	196	57	30.159	29.082
	Vermont	36	29	179	25	86.207	13.966
	West Virginia	5	5	298	1	20	0.336

Table S2. The total and unique (without counting the same species multiple times in separate policy chapters) records of listed species that appear in US Register of Invasives and Introduced Species or the USGS Nonindigenous Aquatic Species databases in invasive species related policy in 21 eastern U.S. states broken apart by major taxonomic groups and the calculated consistency metrics

Taxa	State	Total Species listed in Chapters	Unique invasives listed in Chapters	Unique Invasives in Neighborhood	Number in Common	Among-Neighbor Overlap (%)	Regional Overlap (%)
Plants	Alabama	78	53	124	35	66	28.2
	Connecticut	98	83	155	62	74.7	40
	Delaware	91	65	106	37	56.9	34.9
	Florida	160	99	60	32	32.3	53.3
	Georgia	40	24	170	23	95.8	13.5
	Kentucky	22	18	80	16	88.9	20
	Massachusetts	97	77	172	56	72.7	32.6
	Maryland	72	47	123	31	66	25.2
	Maine	63	52	76	42	80.8	55.3
	Mississippi	51	34	72	24	70.6	33.3
	North Carolina	82	58	91	31	53.4	34.1

	New Hampshire	96	76	98	56	73.7	57.1
	New Jersey	69	38	179	26	68.4	14.5
	New York	161	115	172	62	53.9	36
	Pennsylvania	95	64	195	51	79.7	26.2
	Rhode Island	20	11	175	10	90.9	5.7
	South Carolina	102	48	68	30	62.5	44.1
	Tennessee	53	36	117	26	72.2	22.2
	Virginia	52	38	128	30	78.9	23.4
	Vermont	23	13	177	11	84.6	6.2
	West Virginia	58	46	109	35	76.1	32.1
Invertebr-ates	Alabama	35	19	124	14	73.7	11.3
	Connecticut	14	13	55	10	76.9	18.2
	Delaware	14	7	31	4	57.1	12.9
	Florida	102	62	30	10	16.1	33.3
	Georgia	19	14	96	10	71.4	10.4
	Kentucky	2	1	28	1	100	3.6
	Massachusetts	28	19	54	11	57.9	20.4
	Maryland	21	11	26	6	54.5	23.1
	Maine	21	10	20	5	50	25
	Mississippi	105	69	24	13	18.8	54.2
	North Carolina	38	26	42	19	73.1	45.2
	New Hampshire	32	20	30	13	65	43.3
	New Jersey	28	22	40	11	50	27.5
	New York	59	35	50	18	51.4	36
	Pennsylvania	3	1	54	1	100	1.9
	Rhode Island	32	20	52	14	70	26.9
	South Carolina	29	20	32	11	55	34.4

	Tennessee	14	11	98	10	90.9	10.2
	Virginia	29	17	42	13	76.5	31
	Vermont	15	13	55	12	92.3	21.8
	West Virginia	15	8	22	1	12.5	4.5
Vertebr-ates	Alabama	43	10	46	7	70	15.2
	Connecticut	34	16	42	9	56.3	21.4
	Delaware	42	9	27	7	77.8	25.9
	Florida	83	27	14	4	14.8	28.6
	Georgia	60	9	54	8	88.9	14.8
	Kentucky	77	14	36	11	78.6	30.6
	Massachusetts	3	1	60	0	0	0
	Maryland	78	17	37	16	94.1	43.2
	Maine	27	8	28	6	75	21.4
	Mississippi	141	17	21	10	58.8	47.6
	North Carolina	45	10	42	5	50	11.9
	New Hampshire	122	28	12	8	28.6	66.7
	New Jersey	85	12	33	2	16.7	6.1
	New York	68	30	36	12	40	33.3
	Pennsylvania	11	0	45	0	0	0
	Rhode Island	127	18	42	11	61.1	26.2
	South Carolina	20	11	18	5	45.5	27.8
	Tennessee	25	14	48	11	78.6	22.9
	Virginia	189	30	39	21	70	53.8
	Vermont	29	4	48	4	100	8.3
	West Virginia	5	3	38	1	33.3	2.6

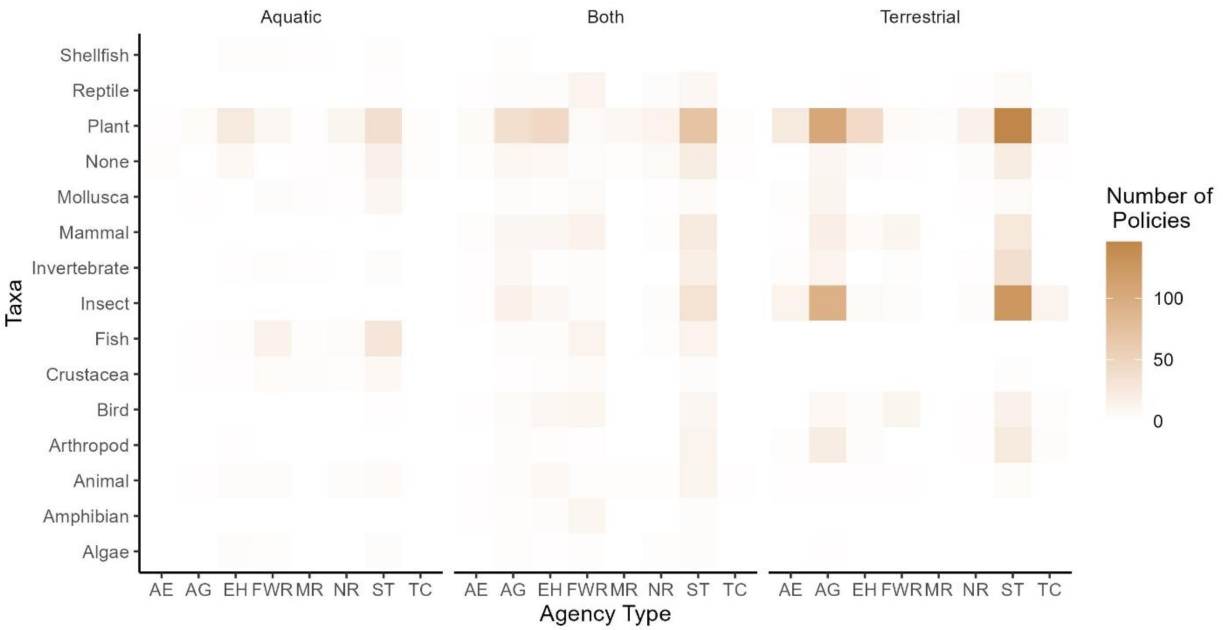

Figure S1: Heat map of the policy chapters for the 21 eastern U.S. states in the policy database relating the number of polices for to statutes (ST, n=479), agricultural, plant, or livestock industries (AG, n=248); wildlife, fisheries, and recreation (FWR, n=84); forestry, mining, and natural resources (NR, n=63); marine resources (MR, n=20); public and environmental health (EH, n=151); transportation, utilities, and commerce (TC, n=29); and admin. education, specific locations, and all other types (AE, n=43).

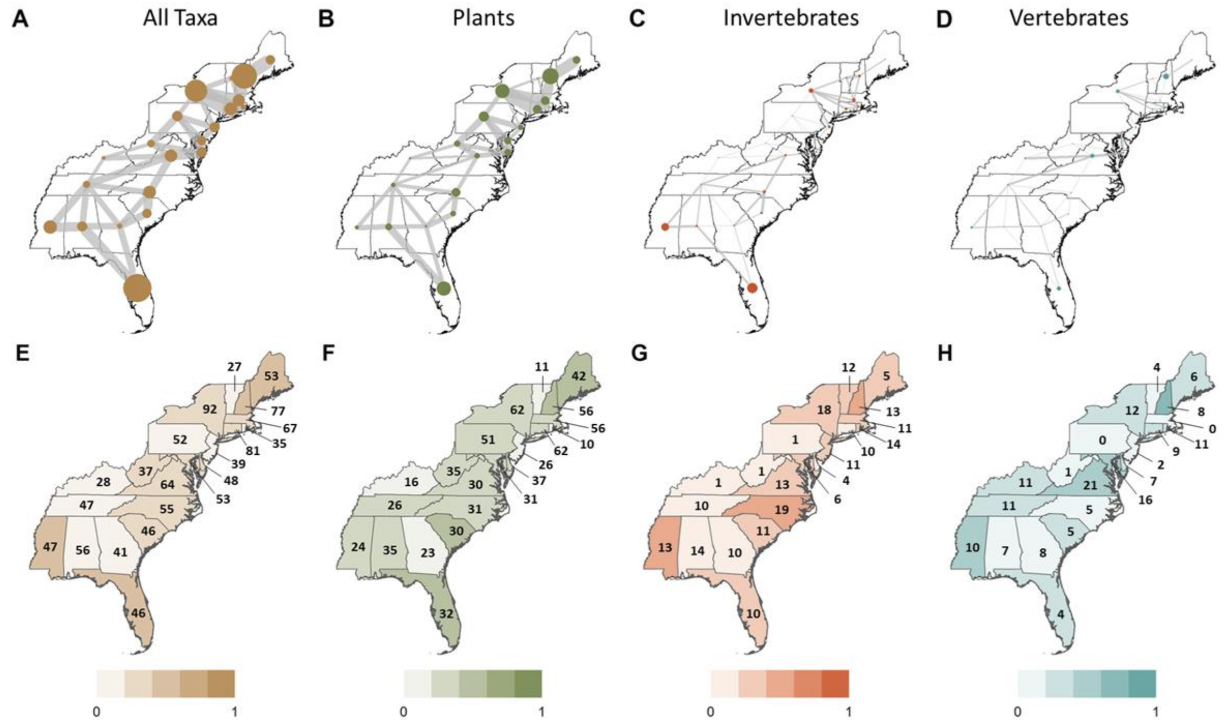

Figure S2: The 21 eastern U.S. states in the policy database and the relative number of policies reference specific taxa (A-D) and relative consistency in policy adoption among states as measured by regional overlap of policies naming species (E-H) where numbers represent the number of species the state has in common with the neighborhood. Number of policies shown in A-H represent a subset of species relative to the full dataset of policy listed species where they must also be referenced in national databases of invasive species (US-RIIS & USGS NAS). Columns represent *all taxa* (A,E), *plants* (B,F), *invertebrates* (C,G), and *vertebrates* (D,H). Network representation of species listed in policies of each state is represented by the relative size of the state centroid node. The width of the edges among these nodes represents the number of species in common among adjacent state pairs. For example, *all taxa* (A) values comprise the total sum of plants, invertebrates, and vertebrates. We see that invertebrates are drastically underrepresented in policy relative to plant species in most states, while a few states disproportionately list many vertebrate species relative to their neighbors. This lack of consistency among policy in adjacent state neighborhoods shows via the intensity of the color among states. However, regional overlap may be high for a focal state if most states in the neighborhood list few species.

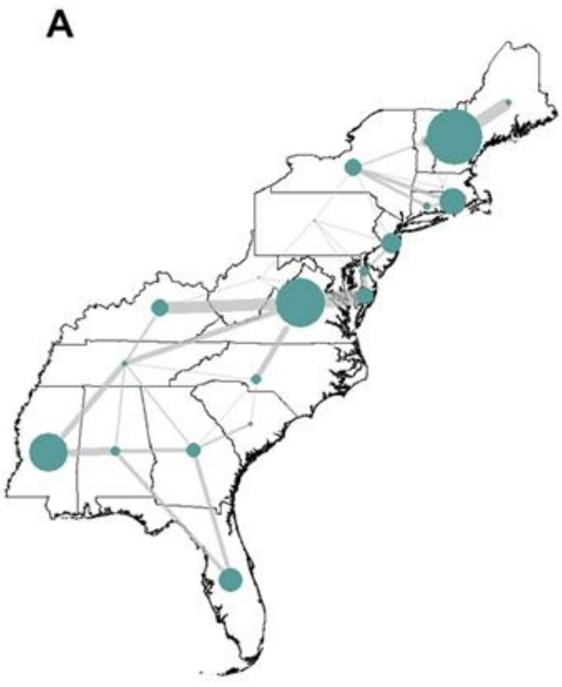

Figure S3: A comparison of A) vertebrate taxa listed in state policy and B) vertebrate taxa listed in state policy that occurs in national invasive species databases (US-RIIS/NAS). Network representation of species listed in policies of each state is represented by the relative size of the state centroid node. The width of the edges among these nodes represents the number of species in common among adjacent state pairs.

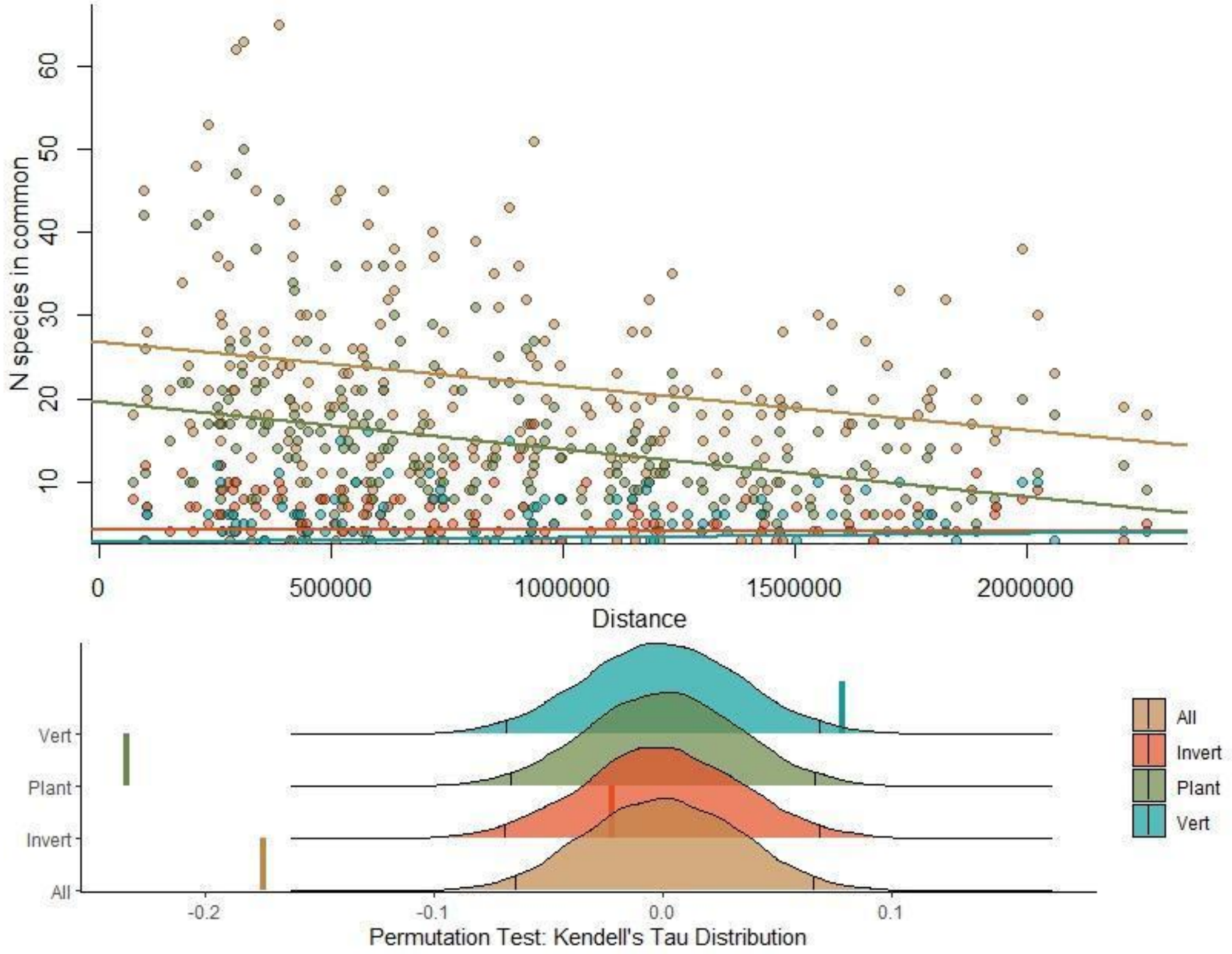

Figure S4: Correlation among pairwise distances and the number of US-RIIS and USGS-NAS identified invasive species a state has named within its policy chapters. As Distance increases, the number decreases. The evidence of this correlation is extremely strong for plants as a taxa. The evidence across all taxa is slightly less but the correlation is significant. Distribution of test statistic values from a permutation test shows that this relationship holds when all taxa are lumped together, but plant taxa has the strongest significance. When vertebrates are considered, there is an extremely weak if significant positive correlation. Vertical colored lines represent the actual value of the Kendall's Tau test statistic and are colored coded to the taxa in the distribution. Vertical black lines within the distribution curve show the 2.5% and 97.5% quantiles needed to find significance.
